# A Vegetable Leaf Disease Identification Model Based on Image-Text Cross-Modal Feature Fusion

**DOI:** 10.3389/fpls.2022.918940

**Published:** 2022-06-24

**Authors:** Xuguang Feng, Chunjiang Zhao, Chunshan Wang, Huarui Wu, Yisheng Miao, Jingjian Zhang

**Affiliations:** ^1^School of Information Science and Technology, Hebei Agricultural University, Baoding, China; ^2^National Engineering Research Center for Information Technology in Agriculture, Beijing, China; ^3^Agriculture Key Laboratory of Digital Village, Ministry of Agriculture and Rural Affairs of the People’s Republic of China, Beijing, China; ^4^Hebei Key Laboratory of Agricultural Big Data, Baoding, China; ^5^Cangzhou Academy of Agriculture and Forestry Sciences, Cangzhou, China

**Keywords:** cross-modal fusion, transformer, few-shot, complex background, disease identification

## Abstract

In view of the differences in appearance and the complex backgrounds of crop diseases, automatic identification of field diseases is an extremely challenging topic in smart agriculture. To address this challenge, a popular approach is to design a Deep Convolutional Neural Network (DCNN) model that extracts visual disease features in the images and then identifies the diseases based on the extracted features. This approach performs well under simple background conditions, but has low accuracy and poor robustness under complex backgrounds. In this paper, an end-to-end disease identification model composed of a disease-spot region detector and a disease classifier (YOLOv5s + BiCMT) was proposed. Specifically, the YOLOv5s network was used to detect the disease-spot regions so as to provide a regional attention mechanism to facilitate the disease identification task of the classifier. For the classifier, a Bidirectional Cross-Modal Transformer (BiCMT) model combining the image and text modal information was constructed, which utilizes the correlation and complementarity between the features of the two modalities to achieve the fusion and recognition of disease features. Meanwhile, the problem of inconsistent lengths among different modal data sequences was solved. Eventually, the YOLOv5s + BiCMT model achieved the optimal results on a small dataset. Its Accuracy, Precision, Sensitivity, and Specificity reached 99.23, 97.37, 97.54, and 99.54%, respectively. This paper proves that the bidirectional cross-modal feature fusion by combining disease images and texts is an effective method to identify vegetable diseases in field environments.

## Introduction

Vegetables are able to provide a variety of vitamins and minerals that are indispensable for the human body, and are one of the essential foods in our daily diet. In recent years, the varieties and frequency of vegetable diseases have continued to increase worldwide with the increase of the vegetable planting area and species. Particularly, due to the changes of global climate and farming system, vegetable diseases have exhibited the characteristics of wide distribution, high disaster frequency and strong suddenness. The spread of diseases has seriously affected the quality and yield of vegetables, not only causing economic losses of billions of dollars each year, but also bringing potential threats to food safety. Therefore, strengthening the disease control measures has become the key to ensuring high yield and high quality of vegetable products.

The premise of disease control is to establish the capability of identifying diseases in a timely and accurate manner. After vegetable leaves are infected with viruses, fungi or bacteria, the external morphological characteristics and internal physiological structure of the infected parts will be subjected to varied changes such as external deformation, fading, curling, rot, discoloration, internal moisture, and pigment content changes. Due to the ambiguity, complexity and similarity of features among different diseases, most of the vegetable growers have difficulty in accurately identifying the disease variety. Consequently, inaccurate disease diagnosis can easily lead to blind application of pesticides, thus missing the best timing for prevention and treatment. Therefore, how to quickly, easily and accurately identify diseases has become an urgent issue to be solved in vegetable production.

In recent years, the advance of computing capacity and the opening of large-scale image datasets have greatly promoted the development of deep learning technology in the field of computer vision recognition. Visual models of various architectures based on the core of convolutional computing have been emerging from time to time, and have been applied in a range of fields with remarkable results. On such a basis, some researchers introduced the deep learning-based vision technology into the research of crop disease identification from three directions, i.e., image classification, target detection, and semantic segmentation, and achieved success to a certain extent.

Disease identification is essentially an image classification task, which is to determine the variety of the disease by building an end-to-end deep learning model to learn the global features of the disease in the images. Dhaka et al. (2021) presented a comparison of pre-processing techniques, CNN models, frameworks, and optimization techniques using leaf images as datasets for plant disease detection and classification. Kawasaki et al. (2015) contributed to the earliest attempt of disease identification by using the CNN model. They trained the CNN model based on 800 cucumber disease images of healthy leaves and two varieties of diseased leaves and achieved an accuracy of 94.9%. Mohanty et al. (2016) compared the performance of AlexNet and GoogleNet on the PlantVillage dataset and found that the average recognition accuracy of GoogleNet was up to 99.34%. In order to further improve the identification performance in complex field environments, Ramcharan et al. (2017) captured 2,756 cassava leaf images in the field environment and applied Inception v3 to identify three varieties of diseases and two types of insect pests; the average accuracy reached 93%. Zhou et al. (2021) adopted progressive learning to guide the model to focus on key feature regions in the disease images; eventually, they achieved a disease identification accuracy of 98.26% in complex backgrounds. Kundu et al. (2021) developed a pearl millet disease detection and classification framework based on the Internet of things and deep transfer learning and reported a classification accuracy of 98.78%.

Unlike the image classification model, the target detection model can not only determine the variety of the disease but also further locate the regions where the disease occurs by learning the local features in disease images. Fuentes et al. (2017) applied the detection framework of Faster-RCNN+VGGNet/ResNet to localize the disease-spot regions of tomato. On a dataset containing 10 varieties of diseases, the mean average precision (mAP) reached 85.98%. Since then, based on the Faster-RCNN framework, researchers have carried out a series of disease detection studies on different crops by modifying the feature extraction module of the backbone network, and achieved satisfactory results (Hong et al., 2018; Wang et al., 2019; Hu et al., 2021; Zhang et al., 2021). Different from the two-stage detection models mentioned above, one-stage detection models can directly predict the category of objects at each position on the feature map without going through the region proposing stage of the two-stage model. Therefore, one-stage-based detection models have also been widely used in disease identification. Maski and Thondiyath (2021) proposed the tiny-YOLOv4 algorithm for detecting plant diseases using mobile agricultural robots, which achieved an mAP up to 99.9% on the papaya ringspot disease. Liu and Wang (2020) proposed an early identification method targeting at the tomato brown spot disease based on the MobileNetv2 YOLOv3 lightweight model. Its F1 score and AP value were both higher than those of Faster-RCNN and SSD models.

Semantic segmentation is to utilize pixel-level features to achieve high-precision separation between disease-spot regions and healthy-leaf regions. The disease-spot regions obtained from semantic segmentation can facilitate the classifier to identify the disease category. In addition, the severity of the disease can be estimated by calculating the ratio of the disease-spot area to the total leaf area. The commonly-used disease image semantic segmentation methods include the fully convolutional segmentation model (Marino et al., 2019; Wspanialy and Moussa, 2020), the encoder-decoder segmentation model (Agarwal et al., 2021; Wu et al., 2021), and etc.

Aforementioned studies have achieved relative success under limited conditions, but disease image recognition in the field environment still faces huge challenges. For example, in the field environment, factors like the complexity of backgrounds, the similarity between features of different diseases, and the great variability of symptoms can significantly increase the difficulty of disease identification. In general, there are two approaches to solve these problems. One is to increase the size of training dataset so that the model can fully capture the features and variations related to each variety of disease (Barbedo, 2018). Due to the complexity and variability of the agricultural production environments, large-scale acquisition and labeling of high-quality datasets is infeasible not only economically but also technically. Another approach is to extract high-accuracy visual features from the images by building a model that is powerful enough for classification prediction (Sladojevic et al., 2016; Thenmozhi and Reddy, 2019). Aim to make the model pay more attention to the key regions of the disease in the image and learn the visual features of the disease from the key areas, the researchers designed a two-stage target detection and recognition network. The main regions in the image containing lesions are first located by the detector, and then these regions are classified by the classifier (Arsenovic et al., 2019; Syed-Ab-Rahman et al., 2022). However, in practice, it is found that there are still limitations in disease identification relying solely on the visual features of leaves.

In this context, the crop disease recognition models based on multimodal learning of images and texts have drawn increasing attention recently. By utilizing the correlation and complementarity between different modalities, multimodal learning can more accurately identify the disease category. The current mainstream multimodal disease identification methods can be roughly divided into two types. The first is a simple probability fusion method, which inputs the data of two modalities into their respective classification models to generate the predicted probabilities, and then combines the predicted probabilities of the two modalities to output the final predicted category. In this method, the data of the two modalities is independent of each other during the feature extraction process, and what are fused are only their classification probabilities. The other one is the feature fusion method, which inputs the data of two modalities into the same model to realize the fusion of the two modalities during the feature learning process. For example, Zhao et al. (2020) added the text modal information (text: geospatial, seasonal, and weather data) into the disease identification model. By encoding the contextual information of the text modality and fusing it with advanced visual features, this method delivered a significantly better performance in crop disease identification than the popular CNN architectures AlexNet and VGG16. Tsai et al. (2019) proposed to use Multimodal Transformer for multimodal feature fusion. The core of this model was a cross-attention layer designed for bidirectional feature fusion, which was able to focus on the interactions between multimodal sequences spanning different time steps and to potentially map from one modality to another. The author’s comprehensive experiment on multimodal sequences suggested that this method had great advantages over the existing methods. Inspired by the multimodal feature fusion method, in the present study, the text modal information was supplemented into the disease identification model so as to construct a Cross-Modal Transformer model that enables the bidirectional fusion of images and texts. The main contributions of this paper are summarized as follows:

1. Aiming at the challenge of vegetable disease diagnosis in the field environment, a data enhancement method was proposed by supplementing disease images with text descriptions in order to improve the disease identification performance of the model.
2. An end-to-end model combining the disease-spot region detector and the disease classifier was proposed. This model allows the classifier to identify the disease category based on the detected disease-spot regions, and its performance was superior to traditional methods.
3. In the disease classification part, a Bidirectional Cross-Modal Transformer Model based on the fusion of image and text modalities was constructed, which further improved the accuracy and robustness of disease identification.

## Materials and Methods

### Data Acquisition

The data used in this study was collected from the Xiaotangshan National Precision Agriculture Demonstration Base. The vegetable images were subjected to a range of interference factors such as similar leaves, ground film, soil, and irrigation pipes, making the backgrounds extremely complex. In view of the influence of the changes in light intensity and illumination angle throughout the day on the accuracy of disease identification, the images were captured in three time periods, i.e., morning (7:00–8:00), noon (11:00–12:00), and evening (17:00–18:00). The dataset consisted of six varieties of diseases (i.e., tomato powdery mildew, tomato early blight, tomato virus disease, cucumber powdery mildew, cucumber virus disease, and cucumber downy mildew), a total of 1,323 images with 1,323 text records. The whole dataset was divided into the training set, the validation set and the test set according to the ratio of 7:2:1 (see Table 1 for the specific number of samples in each set). The text records were natural language descriptions of the disease symptoms shown in the images provided by the growers. Each image and its corresponding text formed an “image-text pair,” as shown in Table 2.

**Table 1:** The number of samples in each set.

Disease class	Number of training images	Number of validation images	Number of testing images
Tomato powdery mildew	112	31	15
Tomato early blight	200	57	28
Tomato virus disease	169	48	24
Cucumber powdery mildew	120	34	17
Cucumber virus disease	169	48	24
Cucumber downy mildew	160	45	22

**Table 2:** Examples of the image-text pair.

Disease category	Text description	Disease image	Disease category	Text description	Disease image
Tomato powdery mildew	Some white powdery spots scatter on the front of tomato leaves.	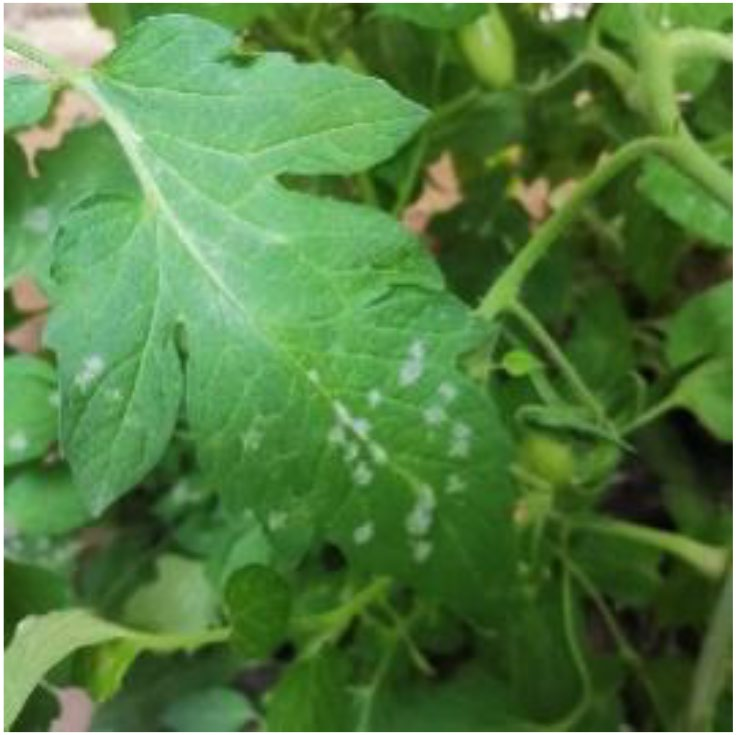	Tomato early blight	Some yellow-brown sunken ring spots are observed on the front of tomato leaves.	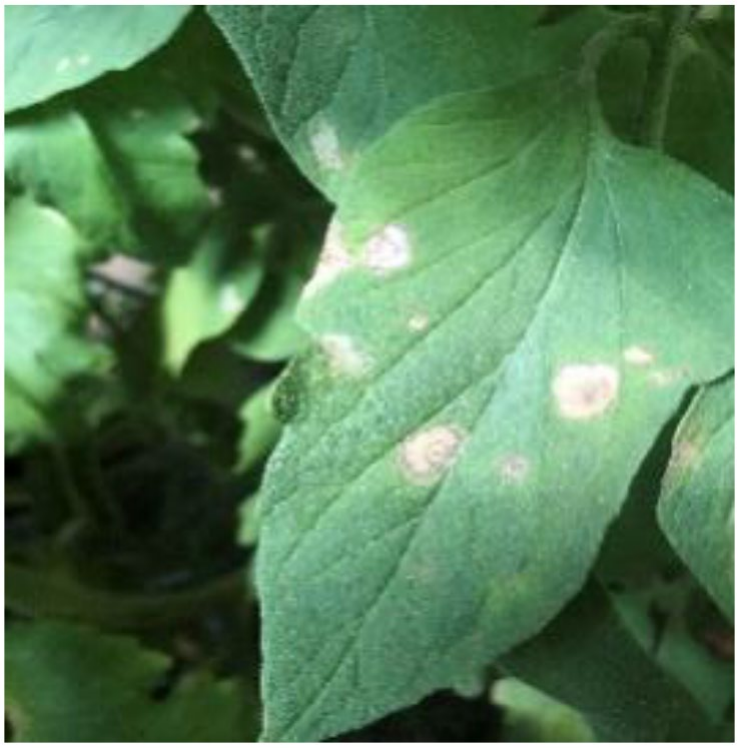
Tomato virus disease	Numerous water-soaked chlorotic spots are observed on tomato leaves.	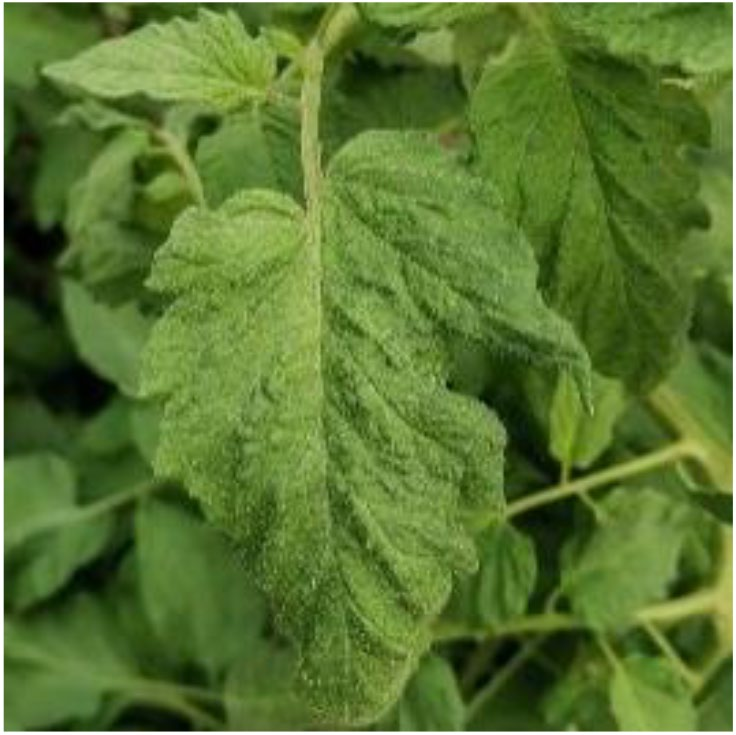	Cucumber powdery mildew	Some white powdery spots scatter on cucumber leaves.	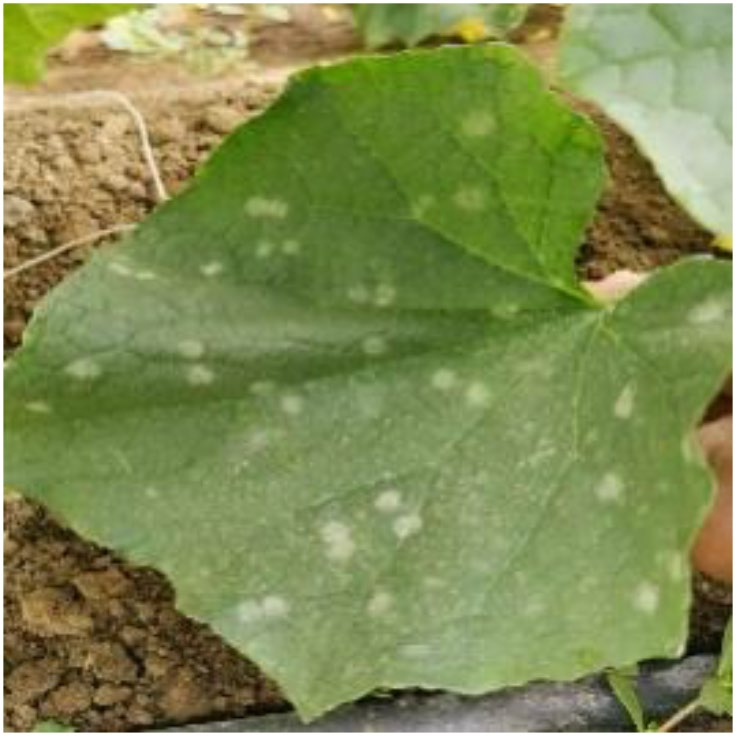
Cucumber virus disease	Numerous chlorotic folded areas are observed on cucumber leaves.	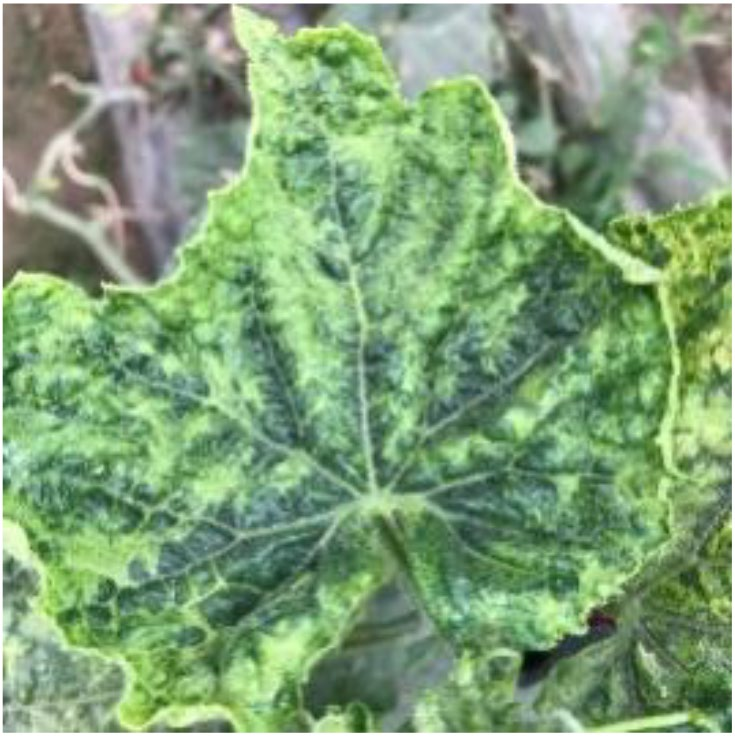	Cucumber downy mildew	Yellow-brown irregular shaped spots scatter on the front of cucumber leaves.	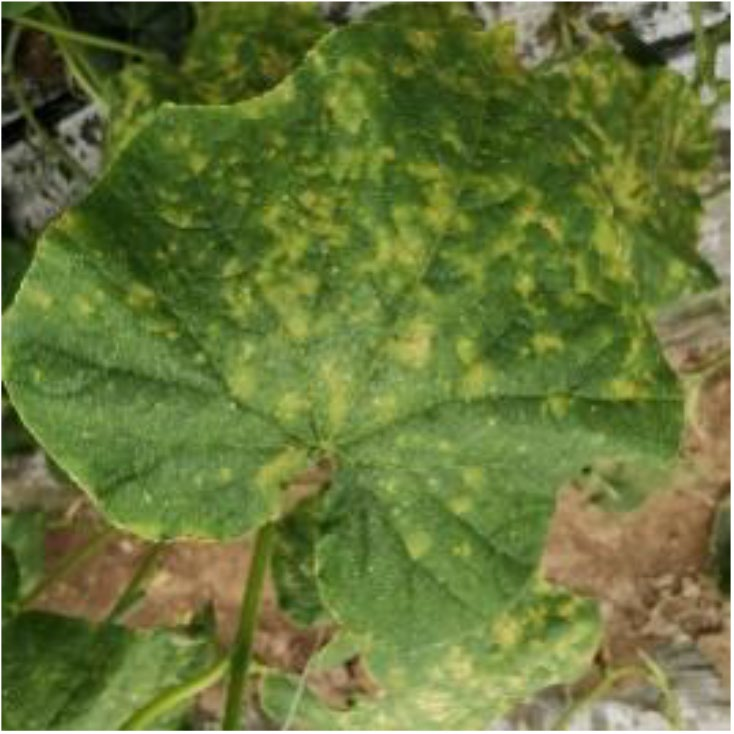

### Data Preprocessing

The image data used for the first-stage detection task was labeled using LabelImg to mark the target regions. For each image, an XML file containing the target category and the coordinate information was generated. Then the labeled images were enhanced for brightness, contrast, and color (see Table 3 for the specific number of images for each variety of disease after enhancement).

**Table 3:** The specific number of images in the dataset.

Disease class	Number of original images	Number of enhanced images
Tomato powdery mildew	158	474
Tomato early blight	285	444
Tomato virus disease	241	482
Cucumber powdery mildew	171	513
Cucumber virus disease	241	482
Cucumber downy mildew	227	420

For the purpose of Chinese text encoding, the Chinese disease descriptions were converted into fixed-length text vectorized encoding sequences by loading the BERT Chinese pre-training model. In this study, the BERT Chinese pre-training model refers to the BERT-wwm, Chinese, base model officially provided by Google, which is trained by the Chinese Wikipedia corpus. The Transformer coding layers of the model were stacked for 12 layers, and the dimension of hidden layers was 768.

## Model Construction

**Figure 1:**
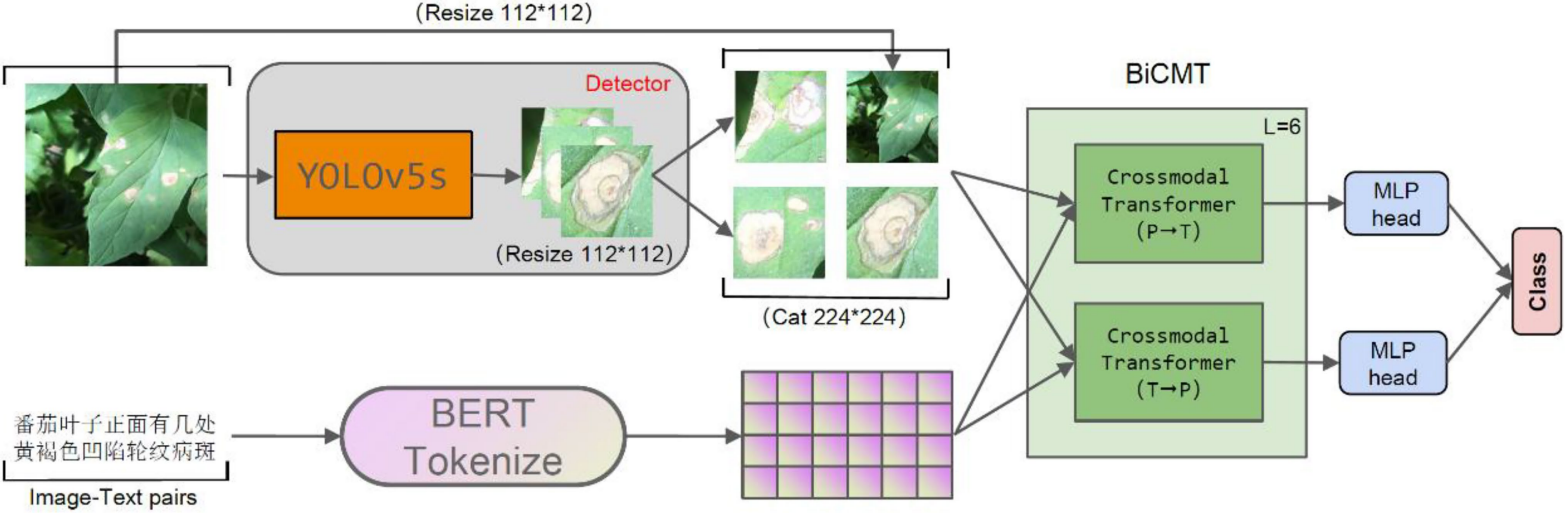
The overall network structure. The input of the model is the “image-text pair.” The detector is YOLOv5s, which is used to detect the disease-spot regions in the images. For text descriptions, Token Embeddings are obtained by loading the BERT Chinese pre-training model. BiCMT is a Bidirectional Cross-Modal Transformer; the two MLP heads of the last BiCMT layer are extracted for classification purpose.

The model proposed in this paper was composed of two stages, i.e., the disease-spot region detector and the disease classifier, which were constructed through two separate training stages. The disease-spot region detector allows the model to focus on the disease-spot regions in the images so as to extract fine-grained disease features while ignoring the interference of factors such as complex backgrounds and irrelevant noises on disease classification. For the first stage of disease-spot region detection, YOLOv5s was chosen as the detection network. For the second stage of disease classification, a Bidirectional Cross-Modal Transformer (BiCMT) network was designed to learn the correlation and complementarity between the image and text modal features through bidirectional cross-modal feature fusion. Owning to more comprehensive and apparent classification features, the performance of the classifier was greatly improved. The overall network structure of the model is shown in Figure 1.

### Fine-Grained Disease-Spot Region Detection

In the first stage, a fine-grained disease-spot region detector was trained for the purpose of extracting discriminative disease-spot regions from the original images. After comparing the current mainstream target detection models, YOLOv5s, which delivered the best performance on the self-collected disease dataset, was chosen as the detector network. Its accuracy in detecting disease-spot regions reached 94%, able to meet the actual detection requirements.

Given the original image $P_{0}$ as the input of the detector, the images containing the disease-spot regions were obtained as the output of the detection network $\left[P_{1},P_{2},\cdots ,P_{n}\right]$ , as shown in Equation (1).


(1) $$\left[P_{1},P_{2},\cdots ,P_{n}\right]=f_{\det}\left(P_{0}\right)$$

where $f_{\det}\left(\cdot \right)$ refers to the disease-spot region detector, and n refers to the number of detected images containing disease-spot regions.

**Figure 2:**
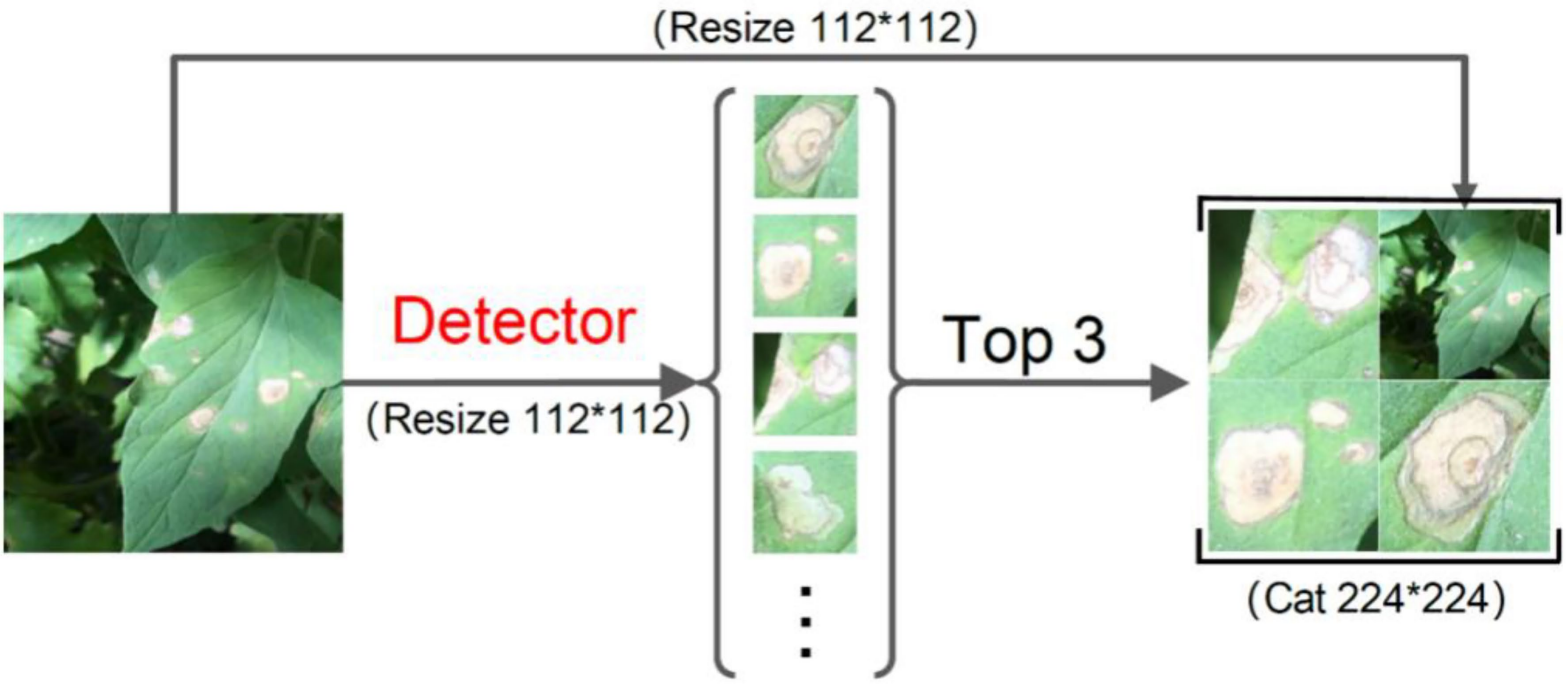
The working principle of fine-grained disease-spot region detection.

On such a basis, the output of the detector was further improved so that it could be used as the input of the classifier. The detected images containing disease-spot regions were sorted in descending order according to the confidence of the detector. The three images with the highest confidence plus 1 original image were selected and scaled to 112 × 112 pixels. Then, the four images were stitched into a composite image of 224 × 224 pixels. The working principle of the fine-grained disease-spot region detector is shown in Figure 2. For the case where the detected images were less than 3, the shortage would be supplemented by randomly enhancing the original image (e.g., rotation, cropping, deformation, and color dithering). For the case where no disease-spot region was detected, the original image would be directly scaled to 224 × 224 pixels for disease classification.

### Disease Classification

In the second stage, the fusion of image and text modal information was used for disease classification. For the image modality, the composite image formed by the fine-grained images containing disease-spot regions and the original image was taken as the input of the classifier. The composite image could provide more comprehensive global features and fine-grained local features of the disease for the classifier. For the text modality, BERT-wwm was used to vectorize the text descriptions. The image modality contains the fine-grained visual feature information of the disease, while the text modality contains the textual descriptions of the key salient features of the disease. It allows the classifier to learn the cross-modal feature mapping matrix of both image and text modal features through the cross-modal attention layer to establish the correlation and complementarity between the two modalities. This approach can obtain better latent feature space embedding, thereby improving the accuracy and robustness of the classifier.

**Figure 3:**
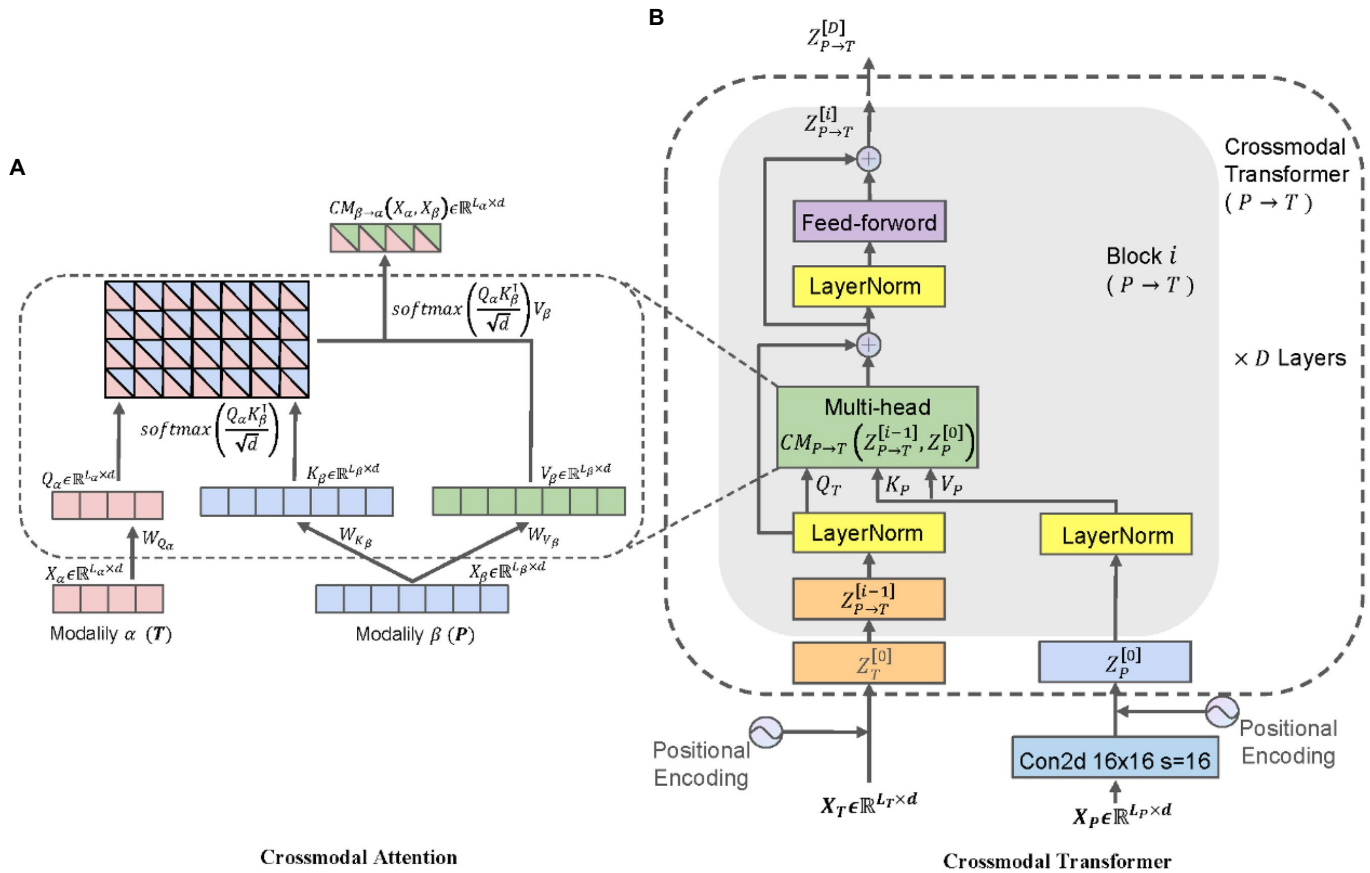
The overall structure of Cross-Modal Transformer.

In this paper, a BiCMT classifier was proposed for modeling unaligned bimodal data sequences, which learns the bidirectional cross-modal mapping sequences from the images and texts by means of feed-forward fusion. Specifically, each cross-modal transformer layer iteratively reinforces the target modality using the low-level features from the source modality by learning the attention across features of two modalities. Therefore, the BiCMT model is built on the two cross-modal mapping branches by stacking multiple cross-modal transformer layers, and finally, the feature vectors of the two cross-modal branches are mapped to a classification layer to predict the disease category. Each cross-modal transformer layer is composed of a cross-modal attention sub-layer and a feed-forward neural network sub-layer, and each sub-layer is normalized with residual connections. The core of this model is the construction of cross-modal attention sub-layers. In the following sections “Cross-Modal Attention”, “Cross-Modal Transformer,” and “Classification Prediction,” each important component of the model will be introduced in detail by taking the mapping from the image modality to the text modality (P→T) as an example, and vice versa. The overall structure of Cross-Modal Transformer is shown in Figure 3.

### Cross-Modal Attention

Let α(T) and β(P) represent two modalities, which are expressed by two unaligned sequences as $X_{\alpha }\epsilon {\mathbb{R}}^{L_{\alpha }\times d}$ and $X_{\beta }\epsilon {\mathbb{R}}^{L_{\beta }\times d}$ , respectively ( L and d refer to the sequence length and feature dimension, respectively). Inspired by machine translation, it is assumed that the fusion of cross-modal information can provide a potential adaptability across different modalities. For example, α→β and β→α represent the bidirectional feature mapping relations between the image and text modalities. The method proposed in this paper aims to realize the feature fusion between two different modalities, i.e., disease images and description texts. By assigning different attention weights to the feature vectors, the feature mapping relation between the source modality β and the target modality α can be established so that the information of these two modalities can be mapped to a strong, unified latent feature space.

Define three attribute values as follows: Querys as $Q_{\alpha }=X_{\alpha }W_{Q_{\alpha }}$ , Keys as $K_{\beta }=X_{\beta }W_{K_{\beta }}$ , Values as $V_{\beta }=X_{\beta }W_{V_{\beta }}$ , where $\left\{W_{Q_{\alpha }},W_{K_{\beta }},W_{V_{\beta }}\right\}\in {\mathbb{R}}^{d\times d}$ represents the weight values. The latent adaptability mapping from β to α can be expressed by the cross-modal attention $Y_{\alpha }\in {\mathbb{R}}^{L_{\alpha }\times d}$ , as shown in Equation (2):


(2) $$\begin{array}{c} Y_{\alpha }=CM_{\beta \to \alpha }\left(X_{\alpha },X_{\beta }\right)\\ =soft\max\left(\frac{Q_{\alpha }K_{\beta }^{Τ}}{\sqrt{d}}\right)V_{\beta }\\ =soft\max\left(\frac{X_{\alpha }W_{Q_{\alpha }}W_{K_{\beta }}^{Τ}X_{\beta }^{Τ}}{\sqrt{d}}\right)X_{\beta }W_{V_{\beta }} \end{array}$$

The sequence length of $Y_{\alpha }$ is expressed as $L_{\alpha }$ , and the feature dimension is expressed as d. $CM_{\beta \to \alpha }\left(\cdot \right)$ represents the cross-modal attention function from the original modality to the target modality. $\sqrt{d}$ is the scaling factor. $\mathrm{softmax}\left(\cdot \right)$ is used to compute the score matrix between two modal feature maps, $\mathrm{softmax}\left(\cdot \right)\in {\mathbb{R}}^{L_{\alpha }\times L_{\beta }}$ . At last, the vector representation after the fusion of cross-modal information can be obtained as the weighted summation of the score matrix and $V_{\beta }$ , as shown in Figure 3A.

### Cross-Modal Transformer

Let $X_{\left\{P,T\right\}}\epsilon {\mathbb{R}}^{L_{\left\{P,T\right\}}\times d_{\left\{P,T\right\}}}$ represent the sequence length and feature dimension of the image and text modalities. In order to maintain the contextual relations for the vectorized encoded sequences, 1D position embeddings were introduced for the two modal vectors, respectively.

The Cross-Modal Transformer was designed based on Cross-Modal Attention, so that the information of the source modality could be mapped to the target modality. The classifier was stacked for six Cross-Modal Transformer layers, and each layer is composed of a Cross-Modal Attention sub-layer and a feed-forward neural network sub-layer. Each sub-layer is normalized with residual connections, as shown in Figure 3B. The Cross-Modal Attention sublayers were configured as mul=12 heads to allow the model to simultaneously take care of features from multiple aspects, and the dimension was fixed as d=768 ( d should be a number divisible by mul ), as shown in Equation (3).


(3) $$\begin{array}{l} Z_{P\to T}^{\left[0\right]}=Z_{T}^{\left[0\right]}\\ {\hat{Z}}_{P\to T}^{\left[i\right]}=CM_{P\to T}^{\left[i\right],mul}\left(LN\left(Z_{P\to T}^{\left[i-1\right]}\right),LN\left(Z_{P}^{\left[0\right]}\right)\right)+LN\left(Z_{P\to T}^{\left[i-1\right]}\right)\\ Z_{P\to T}^{\left[i\right]}=f_{P\to T}^{\left[i\right]}\left(LN\left({\hat{Z}}_{P\to T}^{\left[i\right]}\right)\right)+LN\left({\hat{Z}}_{P\to T}^{\left[i\right]}\right) \end{array}$$

$f_{P\to T}\left(\cdot \right)$ represents the feed-forward neural network sublayer; i represents the *i*-th Cross-Modal Transformer layer, $i\in {\mathbb{R}}^{D}$ ; mul is the number of heads in the Cross-Modal Attention; $LN\left(\cdot \right)$ refers to layer normalization.

### Classification Prediction

In the final classification prediction layer, $Z_{P\to T}$ and $Z_{T\to P}$ extract the cross-modal features from two different directions, respectively. The MLP head of each cross-modal sequence is taken as the classification feature sequence, i.e., $Z_{P\to T}^{{\left[D\right]}^{head}}$ and $Z_{T\to P}^{{\left[D\right]}^{head}}$ . At last, the output probabilities of the two cross-modal classifiers are summed up to further increase the difference between the high-probability classification value and the low-probability classification value, thereby increasing the classification confidence of the Bidirectional Cross-Modal Classifier. The Bidirectional Cross-Modal Classifier can be expressed as Equation (4):


(4) $$Pred_{cls}=soft\max\left(f_{cls}\left(Z_{P\to T}^{{\left[D\right]}^{head}}\right)+f_{cls}\left(Z_{T\to P}^{D^{head}}\right)\right)$$

Where, $Pred_{cls}$ is the predicted category output by the classifier; $\mathrm{softm}ax\left(\cdot \right)$ refers to the softmax activation function; $f_{cls}\left(\cdot \right)$ represents the fully connected layer; $Z_{P\to T}^{{\left[D\right]}^{head}}$ and $Z_{T\to P}^{{\left[D\right]}^{head}}$ represent the cross-modal features learned by the Cross-Modal Transformer in the D layer.

## Results and Discussions

The experiment was carried out in the Ubuntu20.04 environment (processor: Intel Core i9-10920X @ 3.5GHz; memory: 64G; graphics card: NVIDIA GeForce RTX 3090 24G). The deep learning frameworks Pytorch1.7, Python3.8 and Cuda11.0 were used for model building. In the experiment, the batch size was set to 32, and the epoch of all network models was set to 150.

### Evaluation Indicators

Four indicators, i.e., Accuracy, Precision, Sensitivity and Specificity, were used to measure the model performance, as shown in Equations (5–8).


(5) $$Accuracy=\frac{TP+TN}{TP+TN+FP+FN}\times 100\%$$

(6) $$Precision=\frac{TP}{TP+FP}\times 100\%$$

(7) $$Sensitivity=\frac{TP}{TP+FN}\times 100\%$$

(8) $$Specificity=\frac{TN}{FP+TN}\times 100\%$$

TP refers to the number of positive samples that are correctly classified; TN refers to the number of negative samples that are correctly classified; FP refers to the number of positive samples that are mistakenly classified; FN refers to the number of negative samples that are mistakenly classified.

### Comparison of Disease-Spot Region Detectors

In this section, five models of the YOLOv5 series (i.e., YOLOv5l, YOLOv5m, YOLOv5n, YOLOv5s, and YOLOv5x) were chosen for comparison. The depth and width of their backbone networks were set to different values in order to adapt to the detection tasks of different application scenarios. To improve the performance of the detection models, a pre-trained model trained on the ImageNet dataset was loaded during the training process. The batch size of the experiment was set to 16, and the confidence level was set to 0.25. All the disease images were uniformly resized to 640 × 640 pixels. The epoch was set to 300. The experimental results are shown in Table 4.

**Table 4:** Comparison of different YOLOv5 models.

Models	Precision/%	Recall/%	F1/%	mAP/%	Model size(MB)	GFLOPs	FPS
Faster RCNN	66.7	52.8	59.0	59.4	113.6	473.3	23
SSD	56.8	84.4	67.9	68.3	97.7	137.8	51
YOLOv5l	91.6	90.0	90.8	93.4	92.9	108.0	38
YOLOv5m	91.7	90.3	91.0	94.2	42.2	48.1	47
YOLOv5n	91.4	90.2	90.8	93.1	3.9	4.2	54
YOLOv5s	92.3	91.4	91.9	94.3	14.4	15.9	53
YOLOv5x	89.7	91.4	90.5	93.6	173.2	204.3	29

The experimental results show that YOLOv5s had the highest Accuracy, Recall, F1, and mAP among all the models, which were 92.3, 91.4, 91.9, and 94.3%, respectively. Since there were only six disease categories in the dataset and the color, texture and shape of each disease were obvious, YOLOv5s is a suitable choice for this task as it has a smaller model size. Other YOLOv5 models with larger depth and width are prone to the overfitting problem. Therefore, YOLOv5s can well meet the actual detection needs of the first stage.

### Comparison of Disease Identification

In order to demonstrate the superiority of the proposed method, the candidate models were compared from three aspects, namely image modal recognition alone, text modal recognition alone, and cross-modal fusion recognition. The optimizer for all comparative experiments adopted SGD, with a learning rate of $1e^{-4}.$

#### Identification by Image Modality Alone

**Figure 4:**
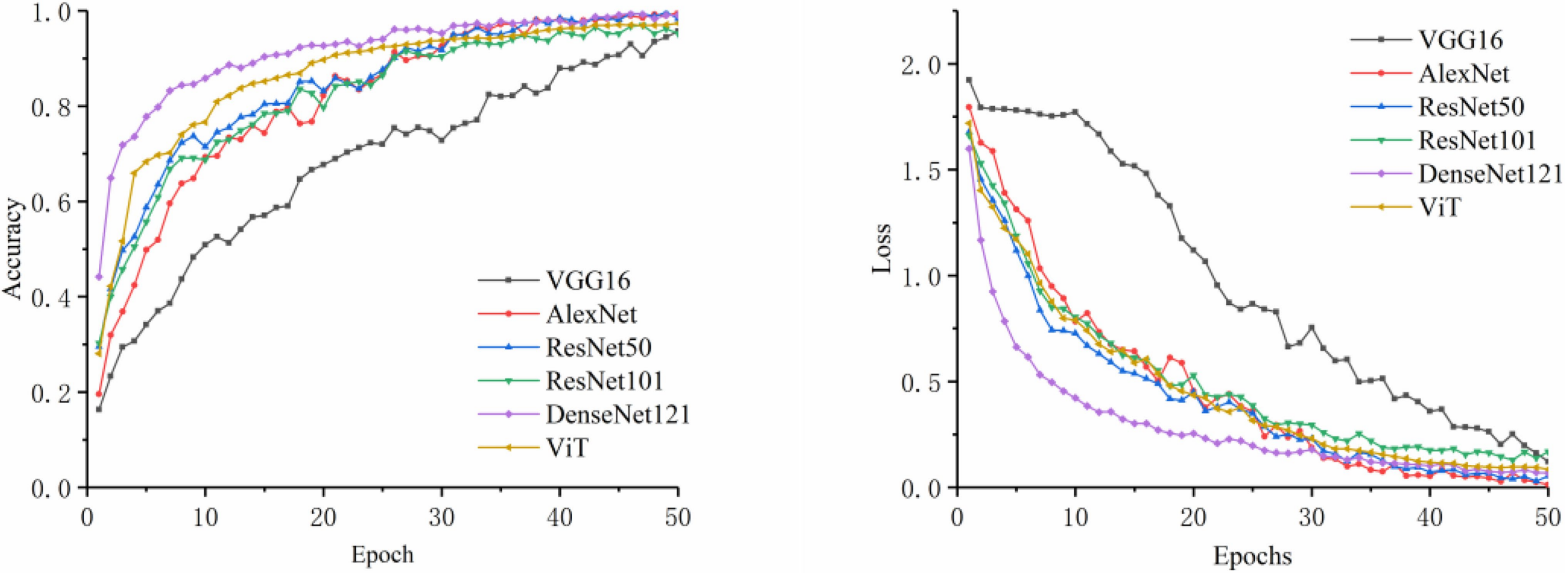
Comparison of accuracy and loss for original image classification.

**Figure 5:**
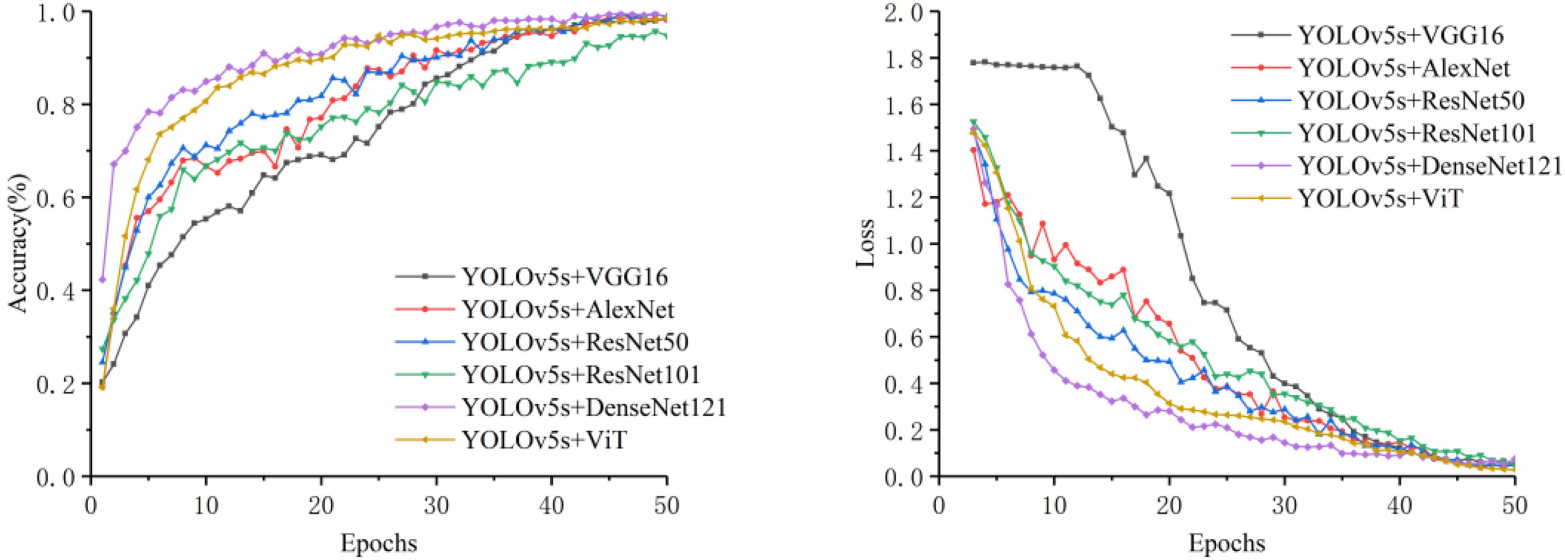
Comparison of accuracy and loss for detection plus classification.

In this section, VGG16, AlexNet, ResNet50, ResNet101, DenseNet121, and ViT (Vision Transformer) were chosen as the image control networks. Two disease classification methods were compared. The first method was to directly identify the disease category from original images. The second method was to use the YOLOv5s model to extract the disease-spot regions from the original images first and then input the composite image containing both the local (images containing disease-spot regions) and global (original image) information into the control network to identify the disease category. The training accuracy and loss curves are shown in Figures 4, 5. The experimental results are shown in Table 5.

**Table 5:** Comparison of image classification results.

Models	Accuracy/%	Precision/%	Sensitivity/%	Specificity/%
VGG16	92.56	77.18	75.17	95.56
AlexNet	95.38	85.63	82.17	97.15
ResNet50	95.38	84.01	83.09	97.24
ResNet101	95.64	85.73	80.04	97.38
DenseNet121	96.67	90.40	87.94	97.96
ViT	95.90	86.90	85.95	97.53
YOLOv5s + VGG16	92.82	84.94	75.67	95.64
YOLOv5s + AlexNet	96.67	89.27	88.81	98.03
YOLOv5s + ResNet50	96.15	87.85	86.62	97.69
YOLOv5s **+** ResNet101	96.15	87.73	86.59	97.66
YOLOv5s + DenseNet121	97.44	91.82	91.24	98.46
YOLOv5s + ViT	96.92	91.44	88.86	98.13

It can be seen from Table 5 that the method of using YOLOv5s to extract the disease-spot regions first before disease classification achieved improvement in all the four indicators (i.e., Accuracy, Precision, Sensitivity, and Specificity) to varying degrees, compared with the direct classification method. It proves that the two-stage method (detector + classifier) is effective for the task of disease identification. This can be explained as follows. The detector is responsible for extracting the disease-spot regions to allow the model to focus on fine-grained local disease features in the images. Then, the classifier further extracts both the local and global disease features so as to complete disease classification. Thus, the detector and classifier are able to concentrate on their respective areas of expertise, with the former assisting the latter to improve the overall classification performance. In the experiment, YOLOv5s + DenseNet121 and YOLOv5s + ViT achieved the highest classification accuracy, i.e., 91.82 and 91.44%, respectively. Transformer shows excellent characteristics in cross modal fusion. Therefore, to enable the model to learn the correlation and complementarity between the information of two modalities so as to further improve the classification performance, a BiCMT model that can fuse the image and text information was designed by choosing ViT as an improved model.

#### Identification by Text Modality Alone

In this paper, text vectorization was realized by two text encoding methods, i.e., the bag-of-words model and the BERT Chinese pre-training model. Then, the experiment was carried out through the Transformer-based text classification model, and the results are shown in Table 6.

**Table 6:** Comparison of the results in the text control group.

Models	Accuracy/%	Precision/%	Sensitivity/%	Specificity/%
BOW+Transformer	95.38	88.20	85.28	97.19
BERT+Transformer	96.46	91.44	91.26	98.07

It can be seen from Table 6 that the disease classification based on texts reinforced by the BERT Chinese pre-training model was superior to the classification reinforced by the bag-of-words model in terms of Accuracy, Precision, Sensitivity and Specificity. The misclassified text descriptions in the test set were usually short in sentence length with vague feature descriptions, and this situation was observed in the classification models with both encoding methods. The bag-of-words model assigns a fixed code to each word in the sentence, regardless of its lexical and grammatical conditions, and each word is independent of each other. The BERT Chinese pre-training model has been optimized for Whole Word Masking training aiming at the Chinese language, so that it can consider the semantic relation between words. After training on a large number of Chinese corpora, each word is mapped to a high-dimensional vector, and the relation between two words can be judged by calculating their cosine distance.

#### Cross-Modal Fusion Identification

**Figure 6:**
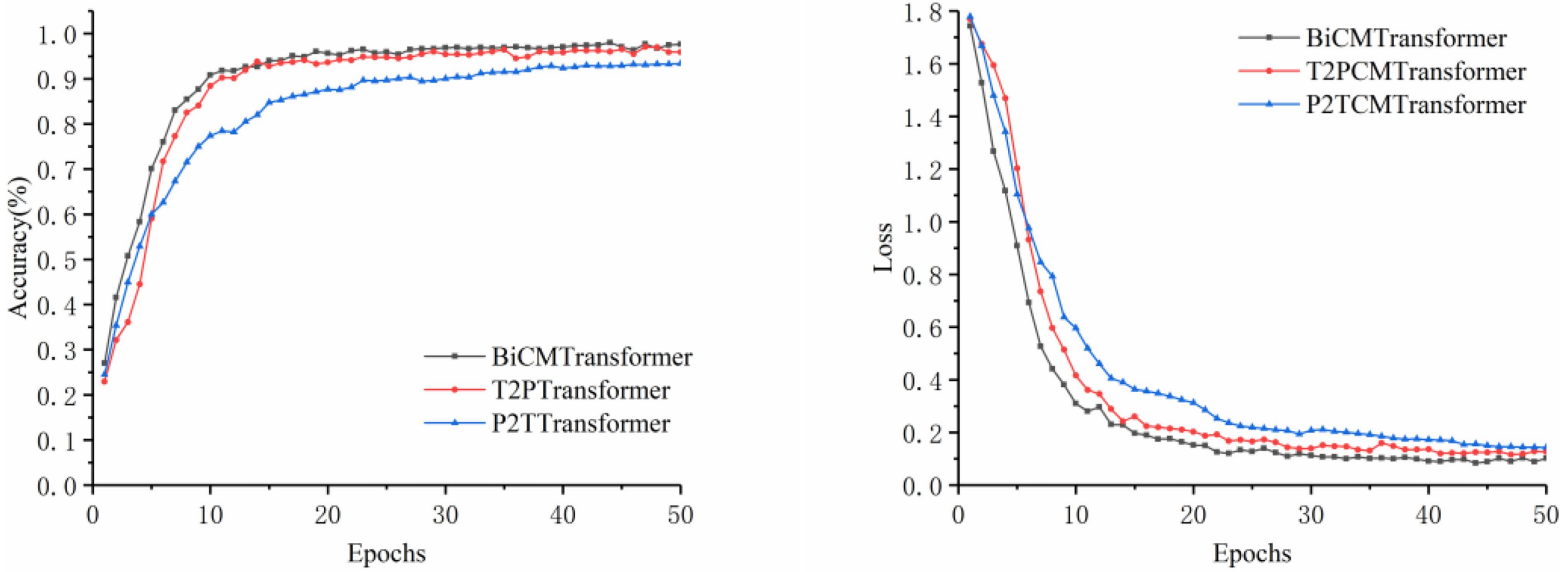
Comparison of accuracy and loss for cross-modal training.

When the image modality or text modality was used for disease identification alone, the identification results would be unsatisfactory once the disease features were not apparent. If the information of the two modalities was used in combination, the fusion of the disease features provided by both modalities could enhance the expression ability of the model, so as to achieve a better identification effect. In this section, three comparative experiments were designed, namely, the Cross-Modal Transformer with the image modality mapping to the text modality (P2TCMT), the Cross-Modal Transformer with the text modality mapping to the image modality (T2PCMT), and the Cross-Modal Transformer with bidirectional mapping of the two modalities (BiCMT). In all experiments, the trained YOLOv5s model was used as the image input of the classifier. The training accuracy and loss curves are shown in Figure 6, and the experimental results are shown in Table 7.

**Table 7:** Comparison of cross-modal training results.

Models	Accuracy/%	Precision/%	Sensitivity/%	Specificity/%
P2TCMT	97.66	92.07	91.82	98.57
T2PCMT	98.21	94.32	94.34	98.93
BiCMT	99.23	97.37	97.54	99.54

As can be seen from Table 7, BiCMT, the cross-modal model with bidirectional mapping between images and texts, achieved the best performance. Its Accuracy, Precision, Sensitivity and Specificity were 99.23, 97.37, 97.54, and 99.54%, respectively. Compared with P2TCMT and T2PCMT (unidirectional cross-modal feature fusion models), BiCMT combined the advantages of the two and could simultaneously learn bidirectional cross-modal fusion information to enhance the correlation and complementarity mapping between the two modalities. Therefore, BiCMT showed a better and more stable training loss, allowing the model to converge faster and more stably to improve the accuracy and robustness of the identification model. The model training accuracy and loss are shown in Figure 6.

**Figure 7:**
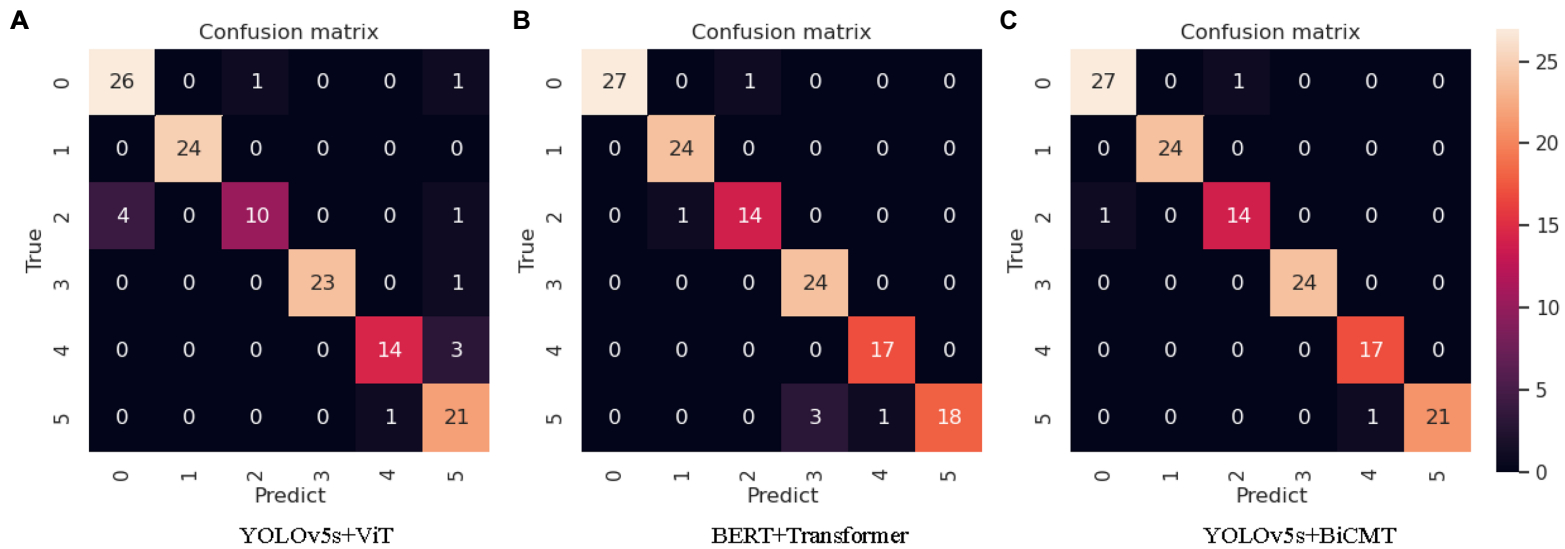
Confusion matrices of the identification results of each model. *Note:* “0” refers to “tomato powdery mildew”; “1” refers to “tomato early blight”; “2” refers to “tomato virus disease”; “3” refers to “cucumber powdery mildew”; “4” refers to “cucumber virus disease”; “5” refers to “cucumber downy mildew.”

In the comparative experiments with images, texts and cross-modal data as the input respectively, the YOLOv5s + BiCMT model based on cross-modal data achieved the best results. The confusion matrix of its identification results is shown in Figure 7C. From the confusion matrices of the three comparative experiments as shown in Figure 7, it can be seen that Figure 7C fully exerted the advantages of cross-modal feature fusion and outperformed Figures 7A,B.

## Discussion

In disease identification, data of a single modality is often subjected to information limitation so that it cannot fully describe the disease features. Compared with single modal data learning, cross-modal data can describe the disease features from multiple angles. Therefore, it is possible to form information complementation through fused representation learning, so as to more comprehensively express the disease features to improve the accuracy of disease identification. In this paper, the BiCMT model was applied to realize the cross-modal fused representation learning between the image and text modal data. The results suggest that the cross-modal fusion model outperformed all single-modal identification models. In terms of cross-modal feature fusion, Zhao et al. (2020) and Wang et al. (2021) applied different models to map the disease features in the images and texts into independent feature spaces respectively, and then fused the probabilities of the identification results of each modality. In this paper, the disease features in the images and texts were directly mapped to a unified feature space so as to realize the feature fusion between cross-modal data. A significant advantage of this method is that the fused representation learning of cross-modal data can be completed in one model, which greatly reduces the training complexity compared to the way of separate learning by different models.

## Conclusion

In this paper, a two-stage identification model (YOLOv5s + BiCMT) composed of a disease-spot region detector and a disease classifier was proposed. First, the detector model was used to extract the disease-spot regions. Then, the detected images containing the disease-spot regions and the original image were stitched into a composite image containing both the global and local disease features. Finally, a BiCMT disease classifier was constructed to classify the disease based on the composite image. BiCMT takes both the image and text modal data as the input in order to utilize the correlation and complementarity between the image features and text features. In the task of vegetable disease identification in complex environments, BiCMT achieved better results than either the image or text single-modal models. Its Accuracy, Precision, Sensitivity and Specificity in the test set reached 99.23, 97.37, 97.54, and 99.54%, respectively. The disease identification method based on the fusion of image and text features has shown obvious advantages. However, in an open environment, cross-modal data is often unbalanced. Specifically, the insufficiency or missing of single-modal information may lead to the “inconsistent strength of modal representation” in cross-modal data, which can affect the final identification results. How to alleviate the adverse effects caused by the lack of modality is an important issue that needs to be further explored in the future.

## Data Availability Statement

The raw data supporting the conclusions of this article will be made available by the authors, without undue reservation.

## Author Contributions

XF: writing–original draft preparation. CZ: methodology and supervision. CW: software and writing–review and editing. HW: writing–review and editing. YM: data curation and validation. JZ: investigation and visualization. All authors contributed to the article and approved the submitted version.

## Funding

This work was supported in part by the National Key Research and Development Program of China, Grant number 2019YFD1101105, the National Natural Science Foundation of China under Grant 62106065, the Hebei Province Key Research and Development Program under Grant 20327402D, the National Technical System of Bulk Vegetable Industry of China under Grant CARS-23-C06, and the Youth Found of Beijing Academy of Agriculture and Forestry Sciences under Grant QNJJ202030.

## Conflict of Interest

The authors declare that the research was conducted in the absence of any commercial or financial relationships that could be construed as a potential conflict of interest.

## Publisher’s Note

All claims expressed in this article are solely those of the authors and do not necessarily represent those of their affiliated organizations, or those of the publisher, the editors and the reviewers. Any product that may be evaluated in this article, or claim that may be made by its manufacturer, is not guaranteed or endorsed by the publisher.

## References

[ref1] Agarwal M. Gupta S. K. Biswas K. K. (2021). A Compressed and Accelerated SegNet for Plant Leaf Disease Segmentation: A Differential Evolution Based Approach[C]//Pacific-Asia Conference on Knowledge Discovery and Data Mining. Springer, Cham, 272–284.

[ref2] Arsenovic M. Karanovic M. Sladojevic S. Anderla A. Stefanovic D. (2019). Solving current limitations of deep learning based approaches for plant disease detection. Symmetry 11:939. doi: 10.3390/sym11070939

[ref3] Barbedo J. G. A. (2018). Impact of dataset size and variety on the effectiveness of deep learning and transfer learning for plant disease classification. Comput. Electron. Agric. 153, 46–53. doi: 10.1016/j.compag.2018.08.013

[ref4] Dhaka V. S. Meena S. V. Rani G. Sinwar D. Kavita K. Ijaz M. F.. (2021). A survey of deep convolutional neural networks applied for prediction of plant leaf diseases. Sensors 21:4749. doi: 10.3390/s21144749, PMID: 34300489 PMC8309553

[ref5] Fuentes A. Yoon S. Kim S. C. Park D. S. (2017). A robust deep-learning-based detector for real-time tomato plant diseases and pests recognition. Sensors 17:2022. doi: 10.3390/s17092022, PMID: 28869539 PMC5620500

[ref6] Hong Q. Quan F. Rui Z. Tianyu L. (2018). Dynamic monitoring of grape leaf disease based on sequential images tracking. Trans. Chin. Soc. Agric. Eng. 34, 167–175. doi: 10.11975/j.issn.1002-6819.2018.17.022

[ref7] Hu G. Wang H. Zhang Y. Wan M. (2021). Detection and severity analysis of tea leaf blight based on deep learning. Comp. Elect. Eng. 90:107023. doi: 10.1016/j.compeleceng.2021.107023

[ref8] Kawasaki Y. Uga H. Kagiwada S. Iyatomi H. (2015). Basic Study of Automated Diagnosis of Viral plant Diseases using Convolutional Neural Networks[C]//International Symposium on Visual Computing. Cham: Springer, 638–645.

[ref9] Kundu N. Rani G. Dhaka V. S. Gupta K. Nayak S. C. Verma S.. (2021). IoT and interpretable machine learning based framework for disease prediction in pearl millet. Sensors 21:5386. doi: 10.3390/s21165386, PMID: 34450827 PMC8397940

[ref10] Liu J. Wang X. (2020). Early recognition of tomato gray leaf spot disease based on MobileNetv2-YOLOv3 model. Plant Methods 16, 1–16. doi: 10.1186/s13007-020-00624-2 32523613 PMC7281931

[ref11] Marino S. Beauseroy P. Smolarz A. (2019). Weakly-supervised learning approach for potato defects segmentation. Eng. Appl. Artif. Intell. 85, 337–346. doi: 10.1016/j.engappai.2019.06.024

[ref12] Maski P. Thondiyath A. (2021). Plant Disease Detection Using Advanced Deep Learning Algorithms: A Case Study of Papaya Ring Spot Disease[C]//2021 6th International Conference on Image, Vision and Computing (ICIVC). IEEE, 49–54.

[ref13] Mohanty S. P. Hughes D. P. Salathé M. (2016). Using deep learning for image-based plant disease detection. Front. Plant Sci. 7:1419. doi: 10.3389/fpls.2016.01419, PMID: 27713752 PMC5032846

[ref14] Ramcharan A. Baranowski K. McCloskey P. Ahmed B. Legg J. Hughes D. P. (2017). Deep learning for image-based cassava disease detection. Front. Plant Sci. 8:1852. doi: 10.3389/fpls.2017.01852, PMID: 29163582 PMC5663696

[ref15] Sladojevic S. Arsenovic M. Anderla A. Culibrk D. Stefanovic D. (2016). Deep neural networks based recognition of plant diseases by leaf image classification. Comput. Intell. Neurosci. 2016, 1–11. doi: 10.1155/2016/3289801, PMID: 27418923 PMC4934169

[ref16] Syed-Ab-Rahman S. F. Hesamian M. H. Prasad M. (2022). Citrus disease detection and classification using end-to-end anchor-based deep learning model. Appl. Intell. 52, 927–938. doi: 10.1007/s10489-021-02452-w

[ref17] Thenmozhi K. Reddy U. S. (2019). Crop pest classification based on deep convolutional neural network and transfer learning. Comput. Electron. Agric. 164:104906. doi: 10.1016/j.compag.2019.104906

[ref18] Tsai Y. H. H. Bai S. Liang P. P. Kolter J. Z. Morency L.-P. Salakhutdinov R. (2019). Multimodal transformer for unaligned multimodal language sequences[C]//Proceedings of the Conference. Association for Computational Linguistics. Meeting. NIH Public Access, 2019: 6558. 10.18653/v1/p19-1656 PMC7195022 32362720

[ref19] Wang Q. Qi F. Sun M. Qu J. Xue J. (2019). Identification of tomato disease types and detection of infected areas based on deep convolutional neural networks and object detection techniques. Comput. Intell. Neurosci. 2019, 1–15. doi: 10.1155/2019/9142753, PMID: 31933623 PMC6942764

[ref20] Wang C. Zhou J. Zhao C. Li J. Teng G. Wu H. (2021). Few-shot vegetable disease recognition model based on image text collaborative representation learning. Comput. Electron. Agric. 184:106098. doi: 10.1016/j.compag.2021.106098

[ref21] Wspanialy P. Moussa M. (2020). A detection and severity estimation system for generic diseases of tomato greenhouse plants. Comput. Electron. Agric. 178:105701. doi: 10.1016/j.compag.2020.105701

[ref22] Wu Z. Yang R. Gao F. Wang W. Fu L. Li R. (2021). Segmentation of abnormal leaves of hydroponic lettuce based on DeepLabV3+ for robotic sorting. Comput. Electron. Agric. 190:106443. doi: 10.1016/j.compag.2021.106443

[ref23] Zhang K. Wu Q. Chen Y. (2021). Detecting soybean leaf disease from synthetic image using multi-feature fusion faster R-CNN. Comput. Electron. Agric. 183:106064. doi: 10.1016/j.compag.2021.106064

[ref24] Zhao Y. Liu L. Xie C. Wang R. Wang F. Bu Y.. (2020). An effective automatic system deployed in agricultural internet of things using multi-context fusion network towards crop disease recognition in the wild. Appl. Soft Comput. 89:106128. doi: 10.1016/j.asoc.2020.106128

[ref25] Zhou J. Li J. Wang C. Wu H. Zhao C. Wang Q. (2021). A vegetable disease recognition model for complex background based on region proposal and progressive learning. Comput. Electron. Agric. 184:106101. doi: 10.1016/j.compag.2021.106101

